# Characteristics of Periodic Ultrasonic Assisted TIG Welding for 2219 Aluminum Alloys

**DOI:** 10.3390/ma12244081

**Published:** 2019-12-06

**Authors:** Xiaoyu Cai, Sanbao Lin, Xianneng Wang, Chunli Yang, Chenglei Fan

**Affiliations:** State Key Laboratory of Advanced Welding and Joining, Harbin Institute of Technology, Harbin 150001, China; xycai@hit.edu.cn (X.C.); yangcl9@hit.edu.cn (C.Y.); fclwh@hit.edu.cn (C.F.)

**Keywords:** ultrasonic assisted welding, 2219 aluminum alloy, periodic ultrasonic vibration

## Abstract

Tungsten inert gas (TIG) arc welding of 2219 aluminum alloy was assisted with a trailing periodic ultrasonic vibration, which was output from a trailing roller behind the welding torch. It was found that the weld appearance was periodically convex due to the periodic input of ultrasonic vibration. With the addition of ultrasonic vibration, the columnar grains in the weld zone transformed to equiaxed grains, so along the longitudinal direction, the equiaxed grains and the columnar grains were alternately distributed due to the periodic ultrasonic vibration. The effects of different ultrasonic powers were investigated. The penetration depth and the amount of the melting metal both increased as the ultrasonic power increased. The coarse precipitated phases in the weld zone tended to disperse uniformly under ultrasonic vibration. Compared with conventional TIG welded joints, the hardness of the weld zone of the ultrasonic assisted TIG welding increased by 8.43%, and the tensile strength increased by 29.02%. The ultrasonic cavitation could decrease the nucleation radius and break the dendrites, which led to the grains’ refinement and the final mechanical properties’ improvement.

## 1. Introduction

Tungsten inert gas (TIG) arc welding is one of the most frequently used welding methods for aluminum alloys. However, its efficiency is low, and the mechanical properties of the welded joint are not good enough due to the poor microstructures. Ultrasonic vibration has been widely used in casting to modify the microstructure through acoustic vibration. Atamanenko et al. [1] used ultrasonic vibration on Al–Cu melts, and it was found that the grain size decreased and that the quality of the castings was improved. Zhang et al. [2] investigated the effects of the high energy ultrasonic field on the microstructures and mechanical properties of A356 alloy, and it was found that the long dendritic silicon phases were broken into pieces and that considerable improvements of the mechanical properties were achieved due to the ultrasonic treatment.

In the welding process, the melting metal will solidify with the decrease of the temperature. Therefore, based on the applications of ultrasonic vibration in casting, ultrasonic energy also has been used in many welding methods. Hu et al. [3] proposed ultrasonic assisted friction stir welding (FSW) for 2219-T6 aluminum alloy, during which ultrasonic vibration acted on the bottom surface of workpieces. It was found that, with the addition of ultrasonic vibration, the hardness distributions in the thickness direction were more homogeneous and that the tensile strength and fracture elongation of the welded joint increased to 359 MPa and 6.7%, respectively. Shah et al. [4] applied ultrasonic assisted resistance spot welding to weld 780 steel and 6061 aluminum alloy. It was found that up to a 300% increase in strength and 150% increase in displacement to failure were achieved.

In arc welding, there are some different modes of ultrasonic addition. Wang et al. [5] used superimposed ultrasonic vibration on the workpiece directly in underwater flux cored arc welding, and an excellent balance of high tensile strength and impact toughness was achieved. Chen et al. [6] also introduced ultrasonic vibration directly on the workpiece during TIG welding for aluminum alloys. It was found that a larger cavitation intensity and a smaller grain size were obtained. However, the test was spot welding, and ultrasonic vibration was not used in the seam welding. Watanabe et al. [7] introduced ultrasonic vibration into the gas metal arc (GMA) welding molten pool through the filler wire and found that the grain size decreased. Based on the acoustic radiation force, a method of adding coaxial ultrasonic vibration in arc welding was proposed by Sun et al. [8] and Fan et al. [9,10]. The arc was compressed, and its energy density increased, which led to the increase of the weld penetration depth. Chen et al. [11] added pulsed coaxial ultrasonic vibration into the molten pool through the arc during GMA welding, and it was found that the microstructures and mechanical properties of the welded joint were improved compared with conventional GMA welding.

Based on the previous studies, in this paper, during the TIG welding process for 2219 aluminum alloy, a periodic ultrasonic vibration acted on the base metal surface through a trailing roller, which kept in touch with the base metal and moved with the welding torch at a constant distance. The weld appearance, microstructures in the weld zone, and the mechanical properties of the welded joint were investigated. The effect of the mechanisms of ultrasonic vibration was analyzed. The 2219 aluminum alloy is mainly used in the manufacturing of the space rockets. The potential application of this research is butt welding for the longitudinal bead of the cylinder tank.

## 2. Experimental Apparatus and Materials

**Figure 1:**
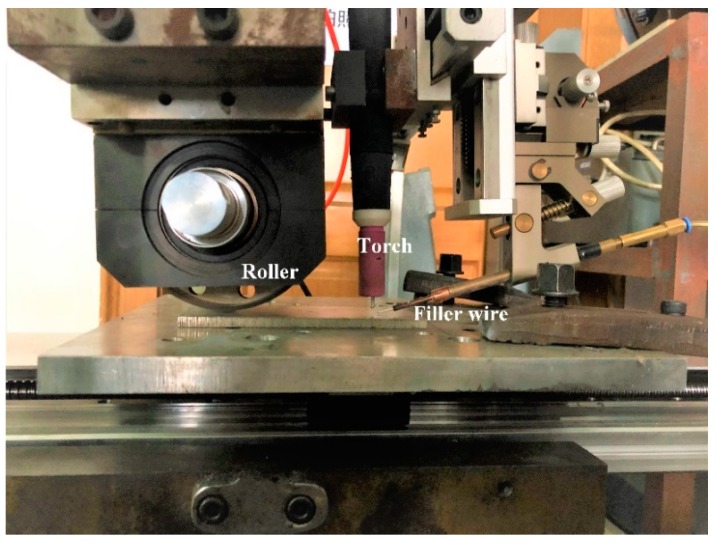
The experimental apparatus.

The main experimental apparatus is presented in Figure 1. A Miller Dynasty 350 TIG welding power source (Miller Electric Manufacturing Co., Appleton, WI, USA) was applied, and it was operated under constant current. The welding torch and the roller were fixed, and the work piece moved at a constant speed. Ultrasonic vibration was output from a contact roller, which rolled on the plate. The distance between the roller and the torch was 90 mm. Ultrasonic vibration was output periodically.

The chemical compositions of the base metal and filler metal are given in Table 1. 2219 aluminum alloy plates with a thickness of 3.5 mm and ER2319 wire with a diameter of 1.2 mm were used as the base metal and filler metal, respectively. Pure argon (99.999%) was used as the shielding gas with a flow rate of 15 L min^−1^. The wire was fed into the molten pool through the TIG arc by the wire feeder. The wire was fed from the front of the arc, and the angle between the plate and the wire was 30°.

## 3. Results and Discussion

### 3.1. Weld Appearance and Penetration Profile

Firstly, bead-on-plate welding processes without filler wire were conducted, and different ultrasonic powers were applied. The main welding parameters are given in Table 2.

**Figure 2:**
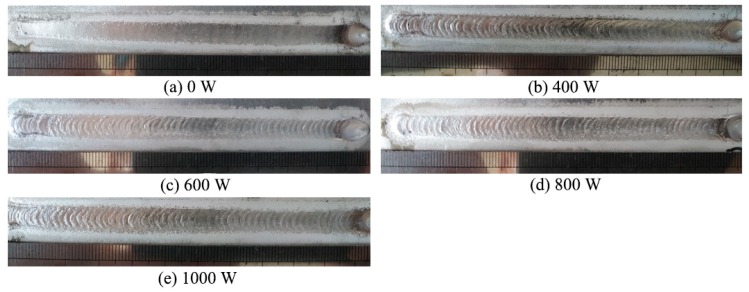
Weld appearances under different ultrasonic powers (T = 0.6 s). Ultrasonic power: (**a**) 0 W; (**b**) 400 W; (**c**) 600 W; (**d**) 800 W; (**e**) 1000 W.

The weld appearance under different ultrasonic powers is presented in Figure 2. It can be seen that, without ultrasonic vibration, the weld’s appearance was smooth. The weld appearance was periodically convex with the addition of ultrasonic vibration. Ultrasonic vibration led to the vibration of the molten pool, which resulted in the convex appearance of the weld surface.

**Figure 3:**
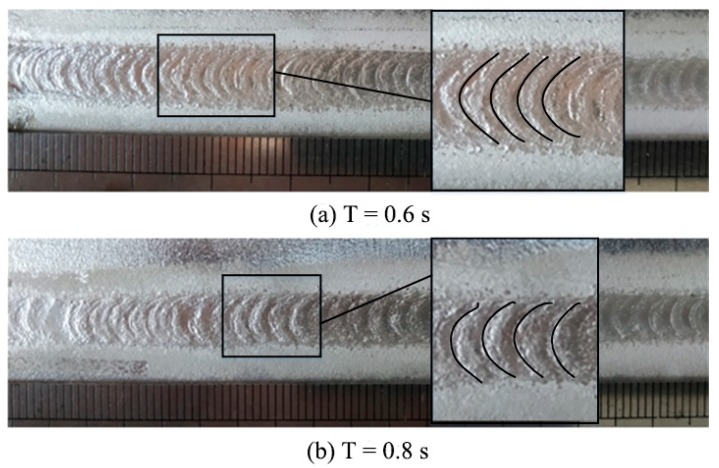
Weld appearances under different ultrasonic periods (ultrasonic power: 600 W). Ultrasonic period: (**a**) 0.6 s; (**b**) 0.8 s.

In order to certify that the ripples were caused by the periodic ultrasonic vibration, different ultrasonic periods were tested. Figure 3 shows the weld appearances under two different ultrasonic periods. The distances between the ripples were measured, and the theoretical distances were calculated (period × welding speed). Table 3 gives the theoretical and measured values, and it can be seen that they were almost equal. Therefore, it can be concluded that the periodic ultrasonic vibration led to the periodic ripples on the weld surface.

**Figure 4:**
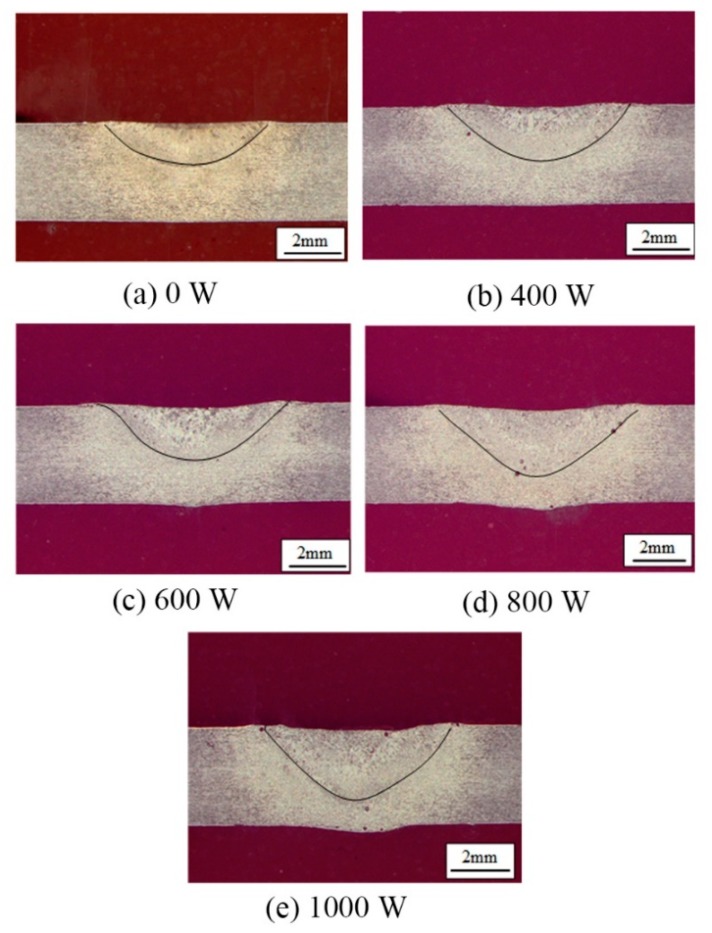
Weld cross-sections under different ultrasonic powers. Ultrasonic power: (**a**) 0 W; (**b**) 400 W; (**c**) 600 W; (**d**) 800 W; (**e**) 1000 W.

**Figure 5:**
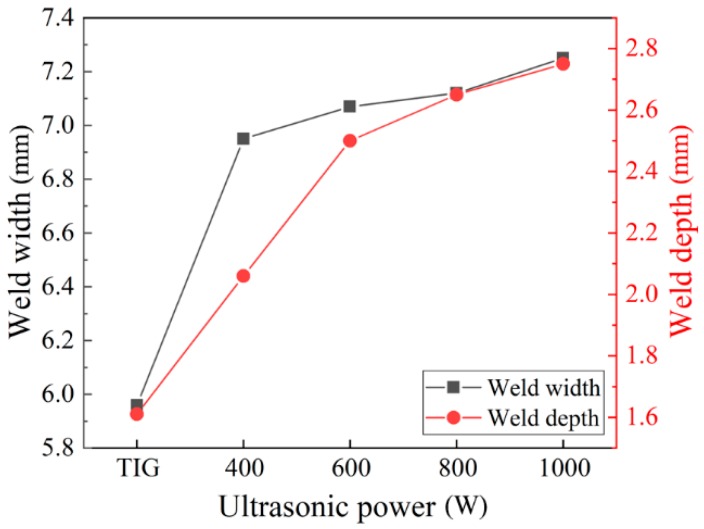
Weld widths and depths under different ultrasonic powers.

**Figure 6:**
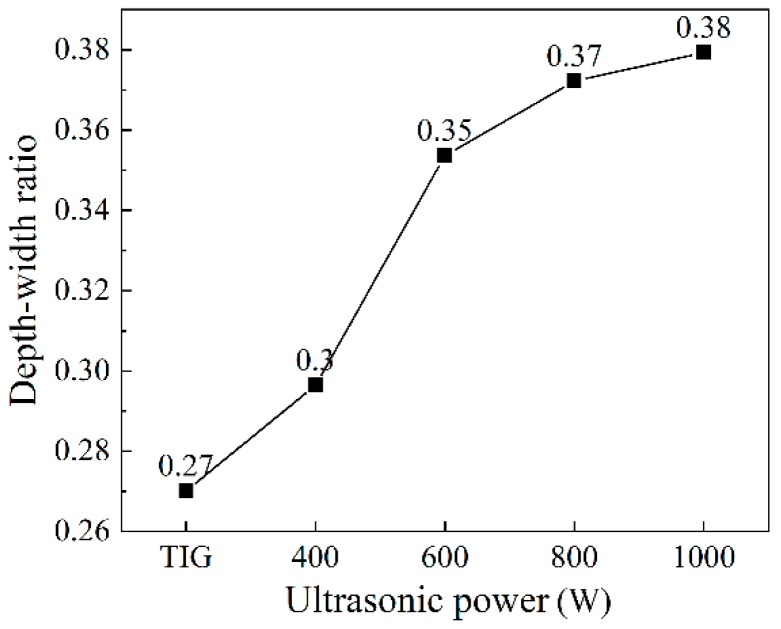
Depth to width ratios under different ultrasonic powers.

**Figure 7:**
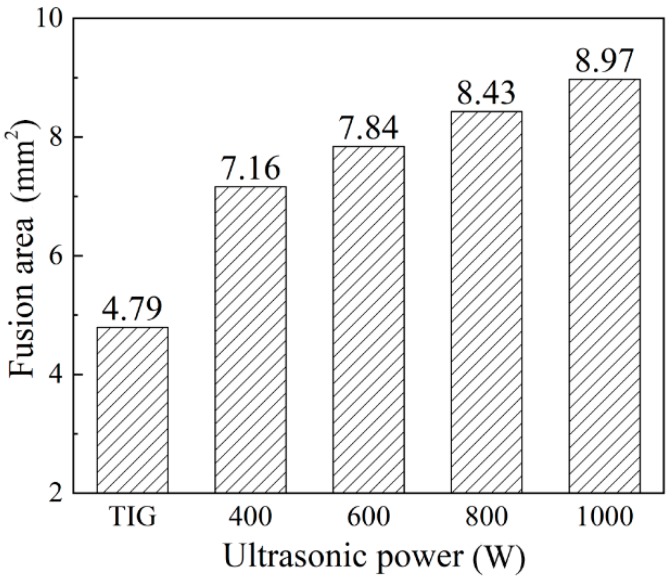
Fusion areas under different ultrasonic powers.

The weld cross-sections are shown in Figure 4. It can be seen that the weld penetration depth (Figure 5), the depth to width ratio (Figure 6), and the fusion area (Figure 7) all increased as the ultrasonic power increased.

Chen et al. [12] analyzed the effect of ultrasonic vibration on the fluid flow of the weld pool by finite element simulation. It was pointed out that the addition of ultrasonic vibration increased the flow velocity of the fluid and made the melting metal around the edge of the molten pool flow upwards, which flows downwards in conventional TIG welding. Therefore, with the addition of the periodic ultrasonic vibration, a periodically convex weld appearance formed. Besides, the increasing ultrasonic power resulted in a faster flow velocity of the high temperature melting metal, which led to the increase of the amount of melting metal.

From the viewpoint of energy transformation, with the addition of ultrasonic vibration, the mechanical energy partly transformed to heat energy, so the total welding energy increased. As the ultrasonic power increased, the total welding energy increased, which led to the increase of the amount of melting metal.

### 3.2. Microstructures and Mechanical Properties

The butt welding process was conducted, and the welding parameters are presented in Table 4. In order to get a full penetration weld, the welding current increased to 170 A.

**Figure 8:**
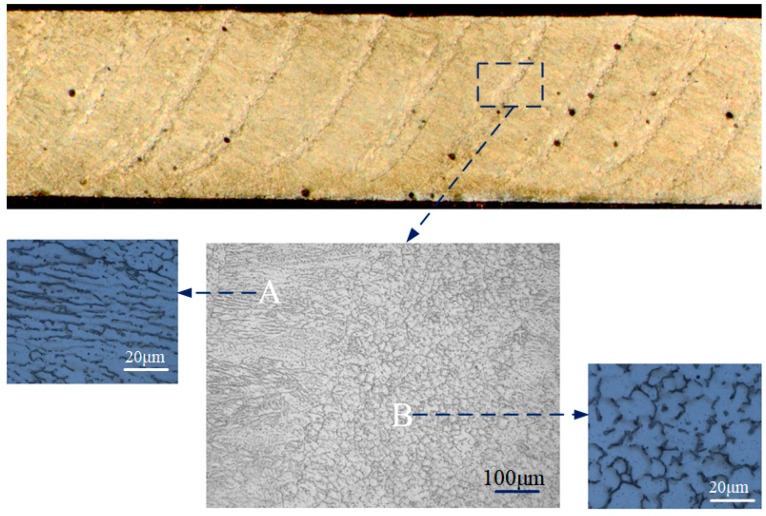
Longitudinal section of the weld.

Due to the addition of periodic ultrasonic vibration, the molten pool was affected by periodic vibration, and the microstructure in the weld zone behaved in a particular manner. The longitudinal section of the weld was observed, as presented in Figure 8. Some curves were distributed along the weld, which resulted from the periodic ultrasonic input. Periodic ultrasonic vibration led to the grains’ morphology changing periodically. The dendrite grains were distributed in the A zone, but the equiaxed grains were distributed in the B zone due to ultrasonic vibration. The B zone behaved as the “curve” in the macro-profile. In the longitudinal direction, ultrasonic vibration transferred into the molten pool through the edge of the molten pool. Because the ultrasonic input was periodic, when ultrasonic vibration was output, the edge of the molten pool was vibrated, and the grains were refined under the vibration, then the B zone with equiaxed grains formed; while ultrasonic vibration was not output, the A zone with dendrite grains formed.

**Figure 9:**
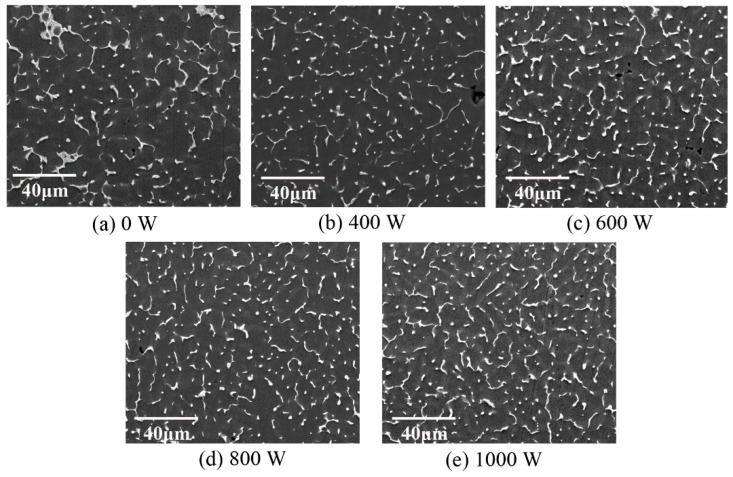
SEM for the weld zone. Ultrasonic power: (**a**) 0 W; (**b**) 400 W; (**c**) 600 W; (**d**) 800 W; (**e**) 1000 W.

As presented in Figure 9, in the weld zone, ultrasonic vibration made the equiaxed grain size decrease and the secondary phase particles were distributed more diffusely. In conventional TIG welding, the massive secondary phase particles were distributed unevenly. Ultrasonic vibration decreased the secondary phase particle size and promoted them to be diffusely distributed. When the ultrasonic power was 400 W and 1000 W, the length of the particle was about 28 μm and 18 μm, respectively. Besides, with the addition of ultrasonic vibration, some secondary phase particles tended to be distributed in the grain interior.

**Figure 10:**
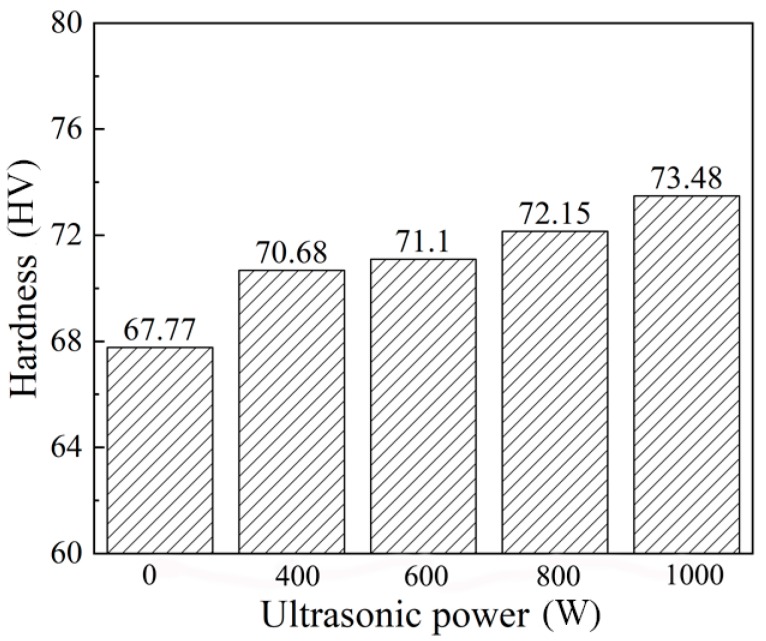
Hardness.

The improvement of the microstructures could enhance the mechanical properties. The precipitation of more and smaller secondary phase particles would contribute to the increase of the hardness of the weld zone and the tensile strength of the welded joint, which was discussed by Cai et al. [13]. As given in Figure 10, the average hardness of the weld zone increased significantly with the increase of the ultrasonic power, and it could be maximally increased by 8.43%.

**Figure 11:**
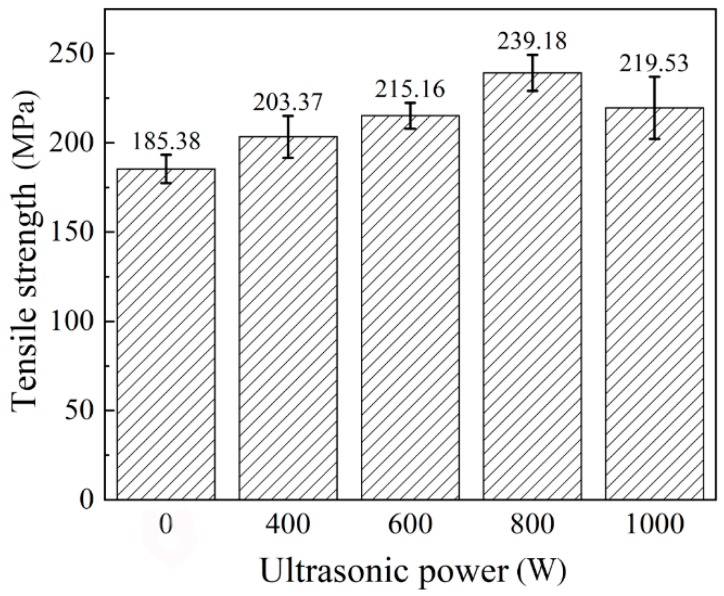
Tensile strength.

**Figure 12:**
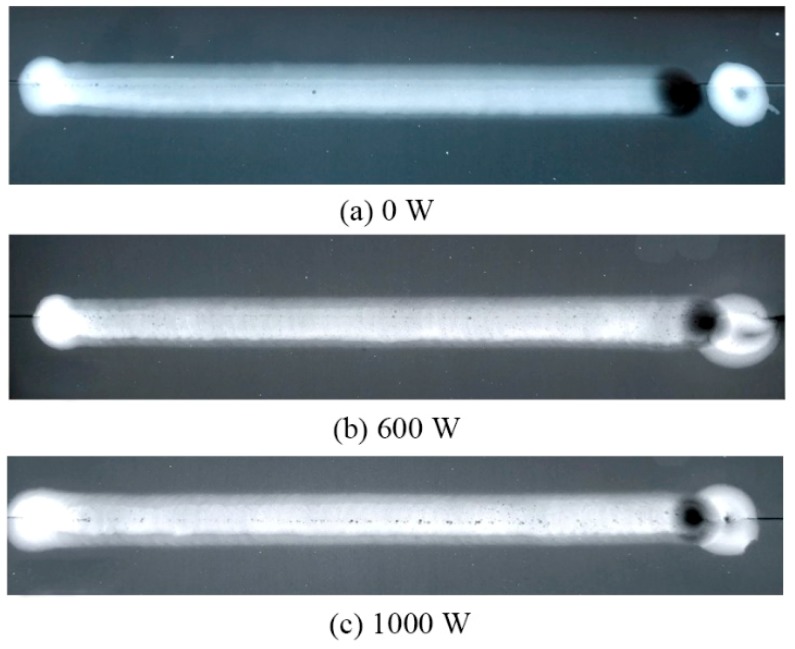
X-ray detection of the joint. Ultrasonic power: (**a**) 0 W; (**b**) 600 W; (**c**) 1000 W.

The tensile strength of the base metal was about 465 MPa. As given in Figure 11, the maximum tensile strength increased by 29.02%. However, it can be seen that when the ultrasonic power increased to 1000 W, the tensile strength decreased. This was because the number of pores in the weld increased, and this led to the decrease of the tensile strength. Figure 12 shows the X-ray detection of the welded joint. Due to the cavitation effect, the addition of the higher power ultrasonic led to the formation of more pores.

### 3.3. Effect Mechanisms of Ultrasonic Vibration on Microstructures’ Refinement

Ultrasonic vibration had two main effects on the grains’ refinement. One was decreasing the critical nucleation radius, and the other one was breaking the dendrites.

The ultrasonic cavitation could result in the formation of cavitation bubbles, and the cavitation bubbles would expand and contract under the acoustic pressure until they collapsed. The collapse of the cavitation bubbles could release a local high pressure (~10^3^ Mpa). As Formula 1 shows, according to the Clausius–Clapeyron relation [14], the increase of the pressure in the liquid metal can increase the crystallization temperature of the melt, which can increase the undercooling of the molten pool’s solidification:

(1) $$\frac{dp}{dT_{m}}=\frac{L_{m}}{T_{m}(V_{L}-V_{S})}$$

where *P* is the pressure; *T_m_* is the crystallization temperature; *L_m_* is the latent heat of fusion; *V_L_* is the liquid phase specific volume; *V_S_* is the solid phase specific volume.

As given in Formula 2, the critical nucleation radius *r_k_* decreased as the undercooling increased. Therefore, the ultrasonic cavitation could promote the decrease of the nucleation radius and the increase of the nucleation rate.

(2) $$r_{k}=\frac{2\sigma_{LS}T_{m}}{L_{m}\Delta T}$$

where *σ_LS_* is surface tension of the solid–liquid interface; Δ*T* is the undercooling.

What is more, the local high pressure induced by the collapse of the cavitation bubbles broke the dendrites and refined the grain. The original coarse dendrites broke into smaller pieces, and the pieces became the new nucleation particles. The number of nucleation particles increased, and the grain size decreased.

**Figure 13:**
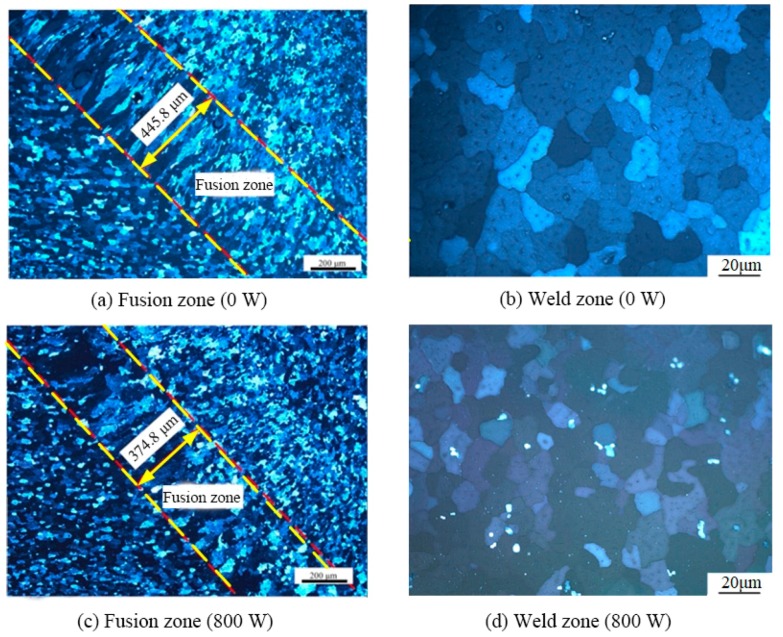
Microstructures of the welded joint for pure aluminum. Ultrasonic power: (**a**,**b**) 0 W; (**c**,**d**) 800 W.

In order to verify that ultrasonic vibration could break the dendrites, a verifying test for pure aluminum (1A99) was carried out. In the pure aluminum, there was almost no second-phase nucleation particle, and it could be explained that the grain refinement resulted from the dendrites’ breaking. A spot welding test was carried out. After extinguishing the arc, during the process of the solidification, ultrasonic vibration was input on the base metal. The welding current was 80 A; the welding time was 5 s; the ultrasonic power was 800 W. Figure 13 points out that the dendrites in the fusion zone were broken; their lengths decreased; and the grain size in the weld zone decreased.

## 4. Conclusions

In this study, a trailing periodic ultrasonic assisted TIG welding process for 2219 aluminum alloy was proposed. The weld formation, microstructures, and mechanical properties of the welded joint were investigated. The main conclusions are summarized as follows.

1. Ultrasonic vibration made the melting metal around the edge of weld pool flow upwards and promoted the flow of the high temperature liquid metal. The periodic input of the ultrasonic energy made the weld’s appearance periodically convex. The weld’s penetration depth and the amount of the melting metal increased as the ultrasonic power increased.

2. The ultrasonic cavitation could promote nucleation and broke the dendrites, which led to the microstructures’ refinement. Under ultrasonic vibration, the columnar grains gradually transformed to equiaxed grains. Periodic crystallization occurred in the weld zone, and the equiaxed grains and the columnar grains were alternately distributed. The precipitated phases in the weld zone tended to be distributed in a dispersed manner.

3. The microstructures improvement enhanced the welded joint’s mechanical properties. Compared with conventional TIG welded joints, with the assistance of ultrasonic vibration, the hardness of the weld zone increased by 8.43%, and the tensile strength of the welded joint increased by 29.02%.

## 5. Perspectives

The addition of ultrasonic vibration can improve the welded joint microstructures and mechanical properties. Some studies can be done in the future:

1. The effects of ultrasonic vibration on the molten pool can be studied by numerical simulation.

2. More kinds of aluminum alloys (Al-Li, Al-Mg, Al-Si, etc.) can be welded by this method, and the welding properties can be investigated.

## Figures and Tables

**Table 1:** Chemical composition of the base metal and filler metal (wt.%).

Element	Cu	Mn	Fe	Ti	V	Zn	Zr	Si	Mg	Al
2319	5.80–6.80	0.20–0.40	0.30	0.02–0.10	0.05–0.15	0.01	0.05–0.15	0.20	0.20	Balance
2219	5.80–6.80	0.20–0.40	0.30	0.10–0.20	0.05–0.15	0.10	0.05–0.15	0.20	0.20	Balance

**Table 2:** Welding parameters of bead-on-plate welding.

Ultrasonic Power (W)	Period (s)	Welding Current (A)	Welding Speed (mm min^−1^)	Arc Length (mm)
0–1000	0.6–0.8	110	200	3

**Table 3:** Theoretical and measured values of the distance between the ripples.

Ultrasonic Period (*s*)	Theoretical Distance (mm)	Measured Distance (mm)	Average (mm)
0.6	2.00	1.95	1.98
		2.01	
		1.98	
0.8	2.67	2.71	2.69
		2.69	
		2.66	

**Table 4:** Welding parameters of bead-on-plate welding.

Period (s)	Welding Current (A)	Welding Speed (mm min^−1^)	Arc Length (mm)	Wire Feed Speed (mm min^−1^)
0.6	170	200	3	200
